# Supplementary material for: Human sand fly challenge elicits saliva-specific innate and type 1-polarized immunity that promotes Leishmania killing
Source: Commun Biol. 2026 May 5;9:933. doi: 10.1038/s42003-026-10130-1 (PMC13350860; doi:10.1038/s42003-026-10130-1)
Supplement: Supplementary file 2 — Supplementary Information [file 42003_2026_10130_MOESM2_ESM.pdf]

SUPPLEMENTARY FIGURES AND TABLE

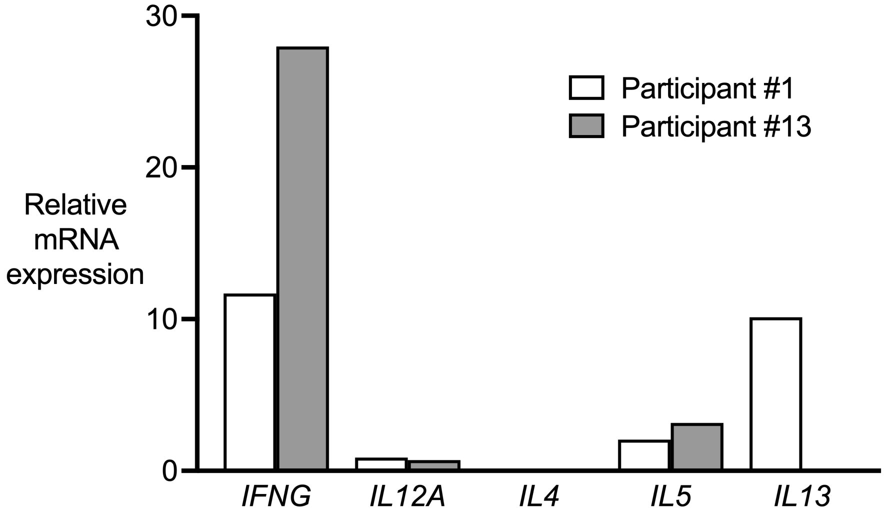

Supplementary Figure 1. Skin cytokine profiles of the delayed-type hypersensitivity response to *Lu. longipalpis* bites. Skin punch biopsies were collected as described in Fig. 3 for measurement of cytokine mRNA expression by quantitative RT-PCR. For each participant, gene expression at the bite site was normalized to expression in normal appearing skin from the contralateral arm.

Supplementary Table 1. PBMC batches by exposure number used for each experiment.

Participant #	Figure 4	Figures 5 and 6	Figure 7
**1**	2	9	7, 8, 9
**2**	2	4	-
**3**	2	9	-
**4**	2	8	-
**5**	2	4	-
**6**	2	6	-
**7**	4	4	-
**8**	4	9	-
**9**	4	5	-
**10**	4	7	-
**11**	4	4	-
**12**	4	5	8
**13**	2	6	9
**14**	2	5	9
**15**	2	5	-

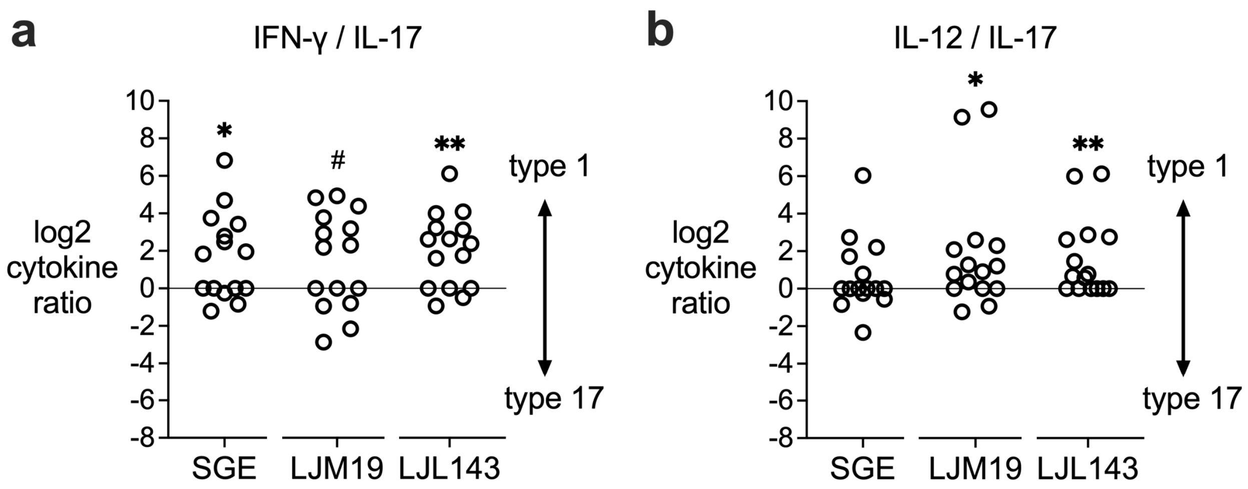

Supplementary Figure 2. LJM19 and LJL143 do not induce a type 17 cytokine response. PBMCs from sand fly-exposed study participants (n = 15) were stimulated with SGE, LJM19, or LJL143. Cytokine concentrations were measured by multiplex bead array and normalized to media-treated, participant-matched cells by background subtraction, as in Fig. 5. Ratios of the type 1 cytokines IFN-γ (**a**) or IL-12 (**b**) to IL-17 were calculated, where log ratios above 0 (solid line) indicate type 1 polarization while log ratios below 0 indicate type 17 polarization relative to media-treated cells, as analyzed by Wilcoxon signed-rank test using a value of zero as the null hypothesis (equal balance of type 1 and type 17 cytokines), # $p < 0.10$, * $p < 0.05$, ** $p < 0.01$.

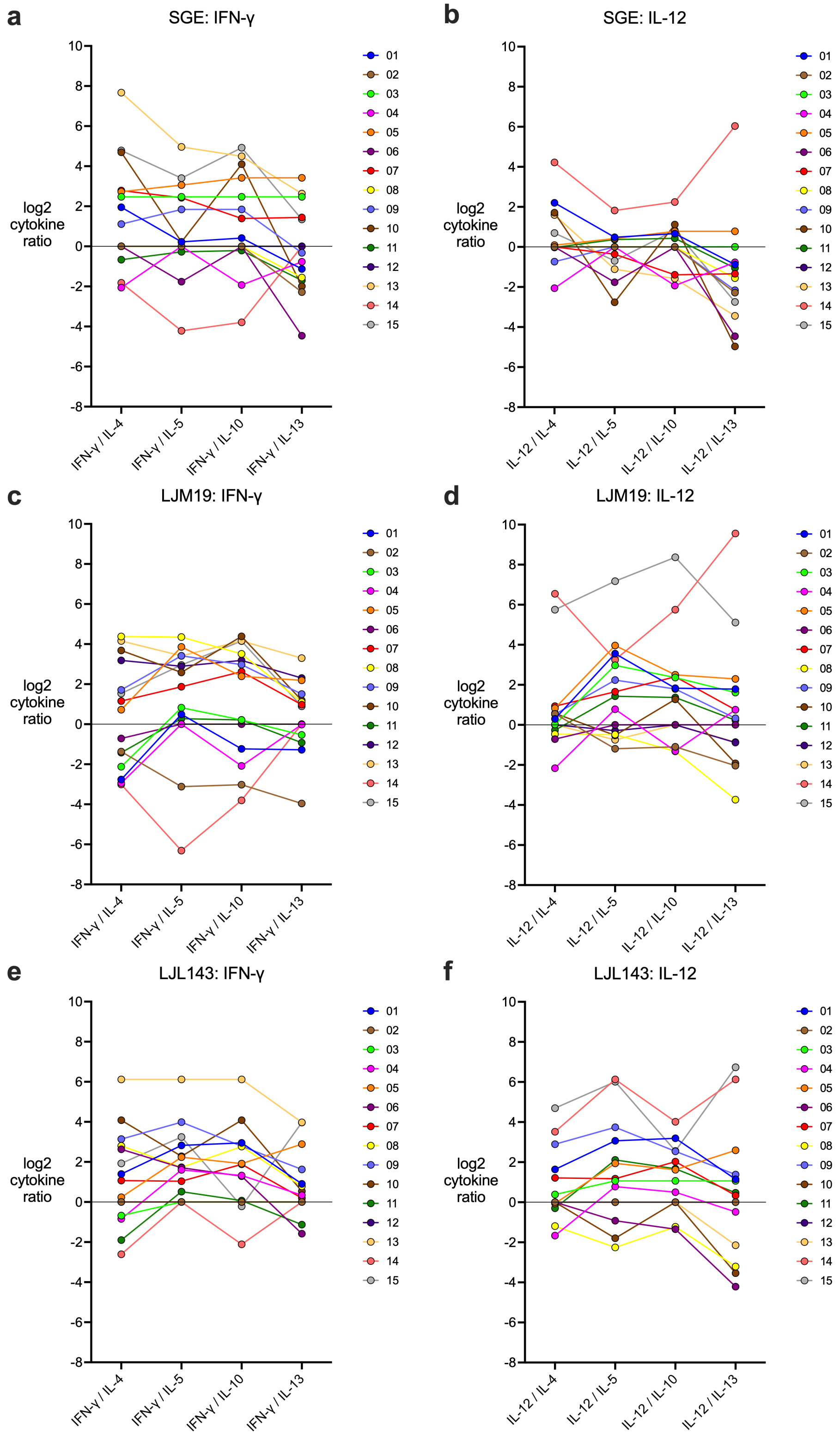

Supplementary Figure 3. Individual type 1/type 2 cytokine response profiles to *Lu. longipalpis* SGE and salivary proteins. Re-plot of data from Fig. 5 of cytokines produced by PBMCs from *Lu. longipalpis* exposed participants following stimulation with SGE (**a, b**), LJM19 (**c, d**), or LJL143 (**e, f**). Each color and line and their connecting points represent a single individual.

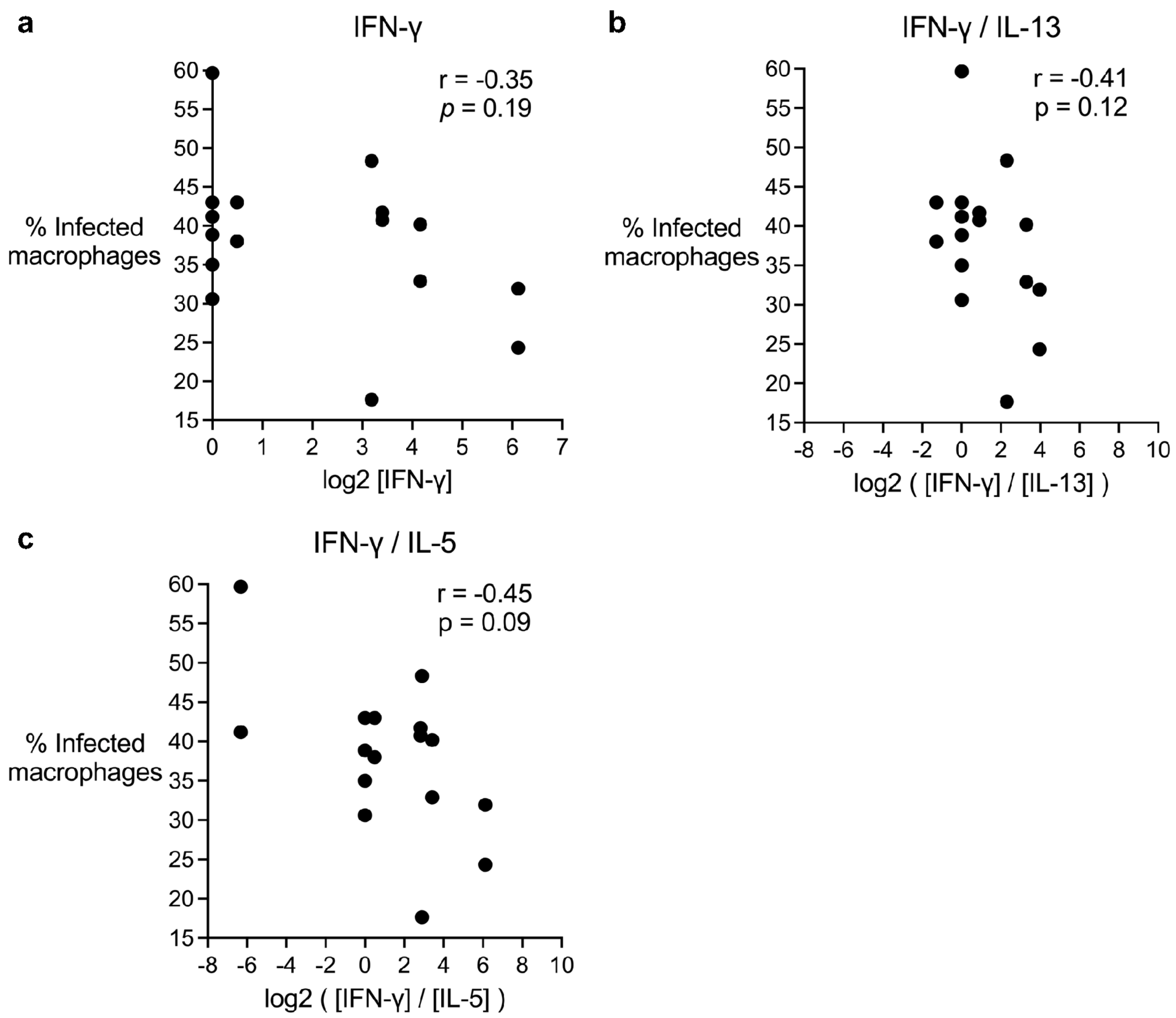

Supplementary Figure 4. Correlation of T_H cytokines with macrophage killing of *Leishmania* parasites. Spearman correlation between IFN-γ alone (**a**), IFN-γ/IL-13 ratio (**b**), or IFN-γ/IL-5 ratio (**c**) as calculated in Fig. 5 and the percentage of infected macrophages for LJM19- and LJL143-treated samples.
