# Supplementary material for: Human sand fly challenge elicits saliva-specific innate and type 1-polarized immunity that promotes Leishmania killing
Source: Commun Biol. 2026 May 5;9:933. doi: 10.1038/s42003-026-10130-1 (PMC13350860; doi:10.1038/s42003-026-10130-1)
Supplement: Supplementary file 3 — Description of Additional Supplementary Files [file 42003_2026_10130_MOESM3_ESM.pdf]

**Description of Additional Supplementary File**

File name: Supplementary Data
Description: Source data underlying all figures and tables are available in the Supplementary Data
