# Supplementary material for: Human sand fly challenge elicits saliva-specific innate and type 1-polarized immunity that promotes Leishmania killing
Source: Commun Biol. 2026 May 5;9:933. doi: 10.1038/s42003-026-10130-1 (PMC13350860; doi:10.1038/s42003-026-10130-1)
Supplement: Supplementary file 5 — Reporting Summary [file 42003_2026_10130_MOESM5_ESM.pdf]

nature portfolio

Corresponding author(s): Joshua R. Lacsina

Last updated by author(s): Apr 13, 2026

Reporting Summary

Nature Portfolio wishes to improve the reproducibility of the work that we publish. This form provides structure for consistency and transparency in reporting. For further information on Nature Portfolio policies, see our Editorial Policies and the Editorial Policy Checklist.

Statistics

For all statistical analyses, confirm that the following items are present in the figure legend, table legend, main text, or Methods section.

n/a	Confirmed	
☐	☒	The exact sample size (*n*) for each experimental group/condition, given as a discrete number and unit of measurement
☐	☒	A statement on whether measurements were taken from distinct samples or whether the same sample was measured repeatedly
☐	☒	The statistical test(s) used AND whether they are one- or two-sided<br>*Only common tests should be described solely by name; describe more complex techniques in the Methods section.*
☐	☒	A description of all covariates tested
☐	☒	A description of any assumptions or corrections, such as tests of normality and adjustment for multiple comparisons
☐	☒	A full description of the statistical parameters including central tendency (e.g. means) or other basic estimates (e.g. regression coefficient) AND variation (e.g. standard deviation) or associated estimates of uncertainty (e.g. confidence intervals)
☐	☒	For null hypothesis testing, the test statistic (e.g. *F*, *t*, *r*) with confidence intervals, effect sizes, degrees of freedom and *P* value noted<br>*Give P values as exact values whenever suitable.*
☒	☐	For Bayesian analysis, information on the choice of priors and Markov chain Monte Carlo settings
☒	☐	For hierarchical and complex designs, identification of the appropriate level for tests and full reporting of outcomes
☐	☒	Estimates of effect sizes (e.g. Cohen's *d*, Pearson's *r*), indicating how they were calculated

Our web collection on statistics for biologists contains articles on many of the points above.

Software and code

Policy information about availability of computer code

Data collection	No software was used for data collection.
Data analysis	Statistical analyses of cytokine data (ELISA and multiplex bead array) and macrophage infection experiments were performed in Prism 10 (version 10.2.0). Analysis of immunohistochemical images was performed with ImageJ (version 1.54m).

For manuscripts utilizing custom algorithms or software that are central to the research but not yet described in published literature, software must be made available to editors and reviewers. We strongly encourage code deposition in a community repository (e.g. GitHub). See the Nature Portfolio guidelines for submitting code & software for further information.

Data

Policy information about availability of data

All manuscripts must include a data availability statement. This statement should provide the following information, where applicable:

- Accession codes, unique identifiers, or web links for publicly available datasets
- A description of any restrictions on data availability
- For clinical datasets or third party data, please ensure that the statement adheres to our policy

The primary dataset for this manuscript is publicly available on figshare at https://doi.org/10.6084/m9.figshare.30397273.

Research involving human participants, their data, or biological material

Policy information about studies with human participants or human data. See also policy information about sex, gender (identity/presentation), and sexual orientation and race, ethnicity and racism.

Reporting on sex and gender	Our study investigated male and female participants, but sex was not specifically tested as a biological variable in the study design due to the limited sample size (n = 15) to evaluate for such an effect. Sex was determined based on self-reporting by the study participants and is reported in Table 1. In our cohort, 3 participants were female and 12 participants were male. All participants were recruited from a military population.
Reporting on race, ethnicity, or other socially relevant groupings	Ethnicity was self-reported by the study participants. All eligible individuals were enrolled in the study regardless of ethnicity. Ethnicity was used as a categorization variable in our study to clarify the limitations of generalizing the study findings to other populations, to inform interpretation of immune variability, and to harmonize across datasets to allow for future cross-study comparisons or meta-analyses. No confounding variables were identified in our analysis.
Population characteristics	Due to the exploratory nature of the study, it was not designed or powered to evaluate the effects of covariates of age, sex, or ethnicity.
Recruitment	Participants recruited were male and female military health care beneficiaries in good health, age 18 to 50 with plans to remain in the Washington, DC area for at least one year to complete all sand fly exposures and study visits. This potentially biases the study due to selective recruitment from a military population, however it is unclear whether or how such a bias would affect our immunological readouts.
Ethics oversight	Walter Reed Army Medical Center and Walter Reed National Military Medical Center (protocol number WR355023)

Note that full information on the approval of the study protocol must also be provided in the manuscript.

Field-specific reporting

Please select the one below that is the best fit for your research. If you are not sure, read the appropriate sections before making your selection.

☒ Life sciences ☐ Behavioural & social sciences ☐ Ecological, evolutionary & environmental sciences

For a reference copy of the document with all sections, see nature.com/documents/nr-reporting-summary-flat.pdf

Life sciences study design

All studies must disclose on these points even when the disclosure is negative.

Sample size	This early-phase mechanistic sand fly human challenge study was exploratory in nature. We chose n=15 to obtain a sample size sufficient to characterize the primary study endpoint of type 1 immune polarization, consistent with WHO human challenge model ethics and early-phase exploratory trial guidance.
Data exclusions	No data were excluded from the analyses.
Replication	For the cytokine ELISA or multiplex cytokine bead array, each assay was run once per sample based on the availability of PBMCs. The sample size of n=15 helped to ensure we captured the heterogeneity of immune responses in the cohort and is comparable to assay sample sizes used in prior human vector challenge studies. The macrophage infection assay was performed with two technical replicates.
Randomization	Randomization was not performed because of the single-arm interventional study design, in which all participants were exposed to sand fly bites via controlled human vector challenge.
Blinding	Blinding was not performed due to the nature of the study objectives.

Reporting for specific materials, systems and methods

We require information from authors about some types of materials, experimental systems and methods used in many studies. Here, indicate whether each material, system or method listed is relevant to your study. If you are not sure if a list item applies to your research, read the appropriate section before selecting a response.

Materials & experimental systems

n/a	Involved in the study
☐	☒ Antibodies
☒	☐ Eukaryotic cell lines
☒	☐ Palaeontology and archaeology
☒	☐ Animals and other organisms
☐	☒ Clinical data
☒	☐ Dual use research of concern
☒	☐ Plants

Methods

n/a	Involved in the study
☒	☐ ChIP-seq
☒	☐ Flow cytometry
☒	☐ MRI-based neuroimaging

Antibodies

Antibodies used	Polyclonals (rabbit anti-human): anti-CD3 (Dako #A0452) and anti-myeloperoxidase (MPO) (Dako #A0398). Monoclonals (mouse anti-human): anti-CD4 (Dako #M7310, clone 4B12), anti-CD8 (Dako #M7103, clone C8/144B), anti-CD20 (Dako #M0755, clone L26), anti-CD68 (Dako #M0814, clone KP1).
Validation	All primary antibodies were validated for immunohistochemistry in tests performed by the manufacturer (Agilent, Dako) and demonstrated in publications cited in the CiteAb database (citeab.com).

Clinical data

Policy information about clinical studies

All manuscripts should comply with the ICMJE guidelines for publication of clinical research and a completed CONSORT checklist must be included with all submissions.

Clinical trial registration	ClinicalTrials.gov # NCT01289977
Study protocol	The full clinical study protocol is available upon request from the corresponding authors.
Data collection	Data were collected at the Walter Reed Army Medical Center and the Walter Reed National Military Medical Center. Recruitment and enrollment in the Lutzomyia-exposed study cohort was performed from October 2010 to March 2011 with completion of clinical data collection in January 2012.
Outcomes	The primary outcome was defined as a type 1 immune response to specific sand fly salivary proteins. This outcome was assessed by stimulating peripheral blood mononuclear cells (PBMCs) from sand fly-exposed participants with sand fly salivary proteins, then measuring the ratios of the concentration of type 1 cytokines (IFN-γ and IL-12) to type 2 cytokines (IL-4, IL-5, IL-10, and IL-13) by multiplex bead array.

Plants

Seed stocks	NA
Novel plant genotypes	NA
Authentication	NA
