# Supplementary material for: Human sand fly challenge elicits saliva-specific innate and type 1-polarized immunity that promotes Leishmania killing
Source: Commun Biol. 2026 May 5;9:933. doi: 10.1038/s42003-026-10130-1 (PMC13350860; doi:10.1038/s42003-026-10130-1)
Supplement: Supplementary file 1 — Transparent Peer Review file [file 42003_2026_10130_MOESM1_ESM.pdf]

nature portfolio

Peer Review File

Human sand fly challenge elicits saliva-specific innate and type 1-polarized immunity that promotes *Leishmania* killing

Corresponding Author: Dr Joshua Lacsina

This file contains all reviewer reports in order by version, followed by all author rebuttals in order by version.

Version 0:

Reviewer comments:

Reviewer #1

(Remarks to the Author)
This work is relevant to the field of leishmaniasis and provides useful evidence on the immune responses generated after repeated exposure of Lu. longipalpis bites in humans. Despite limitations aknowledged by the authors, the results contribute to the understanding of immunity generated by this sand fly species salivary proteins, identifiyng potential vaccine candidates and raising new questions.
The paper is well-written, the methodology fully described and the statistical analyses are valid.

I have no comments on the manuscript, just suggest to review the use of italics for the vector species throughout the document.

Reviewer #2

(Remarks to the Author)
The study on controlled exposure to sand fly bites in humans is tremendous, providing many meaningful findings, and I found this worthy for being shared to a wide range of authors working in immunology, parasitology and entomology. At the same time, the study somewhat struggles with showing statistical significance to draw conclusions the authors would like to deliver. I understand the difficulty in having clear-cut data on clinical subjects, but careful interpretations and discussions of data as well as statements of limitations would help readers aware of the significance of the study, so the authors are encouraged to consider comments provided below.

L169: 'The IHC data also suggest that CD4–CD8– T cells (termed double negative or DN T cells) may play a role in the DTH response to sand fly bites.' Though this claim is partly reasonable according to previous papers, technical problem /sensitivity issue on CD4 staining by IHC cannot be ruled out. For example, Ref. 20 surely describes the presence of DN T cells in CL lesions, the proportion is much less than CD4+ T cells. Ref. 21 reports a higher proportion of DN T cells, whereas CD4+ T cells still account for one third of T cell population. As an accumulation of CD4+ T cells is a hallmark of DTH, careful interpretation of the data is necessary if the authors would like to claim the observed reaction is truly DTH. One of the potential solutions which can be done using obtained samples is to assess CD4 gene expression using RNA isolated from the biopsy samples. If performing additional experiments is difficult, at least limitations need to be discussed in the paragraph starting on L317.

Fig. 4a and 5a: Though I understand a difference in measurement methods, why do IFN-γ levels differ this much (almost two orders when medians are compared) between the two experiments?

Fig. 5: It is new to me to interpret log2 cytokine concentration ratio of 0 as a boarder for Th1/Th2. I do not think this is a well-accepted method. In general, IFN-γ shows a higher concentration than IL-4 or IL-5, and if sole concentration ratio is used, it naturally shows a trend of 'Th1-biased immunity' in many cases. Besides, IFN-γ/IL-17 is no longer Th1/Th2.
I am also confused on the authors' intention on the figure. If the intention is to claim 'LJM19 and LJL143 induce a TH1-

polarized cytokine response (L194)', the authors need to do comparison between those defined antigens and SGE, as they did for Fig. 5a. Despite that, Fig. 5b-g do not involve statistical analyses among antigens. Proper analyses are required to draw conclusions the authors would like to claim.

Fig. 7b and Fig. S3: Can you enumerate 'the number of amastigotes per 100 macrophages (both infected and uninfected)' and see if there is any statistical difference between the unexposed and exposed? This is proposed because no statistical difference was observed between the unexposed and exposed in Fig. 7b or Fig. S3; 'a narrower data spread' (L272) is not very helpful to evoke the presence of antigen-specific Th1 cells limiting parasite infection. If none of the analysis does not work, limitations are better to be discussed more carefully in the Discussion section. For example, L362 says 'our data from human PBMCs show that LJL143 promotes the killing of 362 L. infantum by infected macrophages ex vivo.' in the context of vaccines, i.e., antigen-specific acquired immunity. But, the data say the parasite-killing activity of LJL143 is not depended on antigen-specific acquired immunity. This kind of overinterpretation can also be found elsewhere (L399 – 411).

Reviewer #3

(Remarks to the Author)
Summary
This study established a cohort of 15 sand fly saliva immunized humans, previously unexposed, through repeated exposure over the course of 46 weeks. This resulted in a cohort with salivary protein reactive cellular response and presumed skin tissue resident immune seeded environment. The authors assayed the site of skin exposure for high level skin infiltration post challenge in 2 subjects, which showed highly variable infiltrate, but both indicated a significant presence of double negative T cells in the skin, which is an interesting finding to follow up on as DN T cells are underexplored. The authors also assays PBMCs restimulated with salivary proteins to identify the two specific proteins that induce the strongest Type 1 polarized response. These proteins are able to induce antigen-specific IFNy secretion at levels similar to whole salivary gland extract. This is a considerable achievement as bulk SGE has variable pleiotropic and non-specific effects on local cells whereas use of a specific recombinant protein has more potential for therapeutic development and controlled testing of effects. Finally, a coculture experiment using autologous parasite infected macrophages and salivary protein stimulated PBMCs shows that salivary protein stimulated PBMCs results in significantly fewer infected macrophages compared to unstimulated PBMCs. This finding is to be determined if it is influenced on pre-immunization with salivary proteins or not. The study lacks flow cytometry on the antigen stimulated PBMCs to phenotype relevant cytokine secreting cell type lineage and memory/effector phenotype. The study also lacks unstimulated controls, instead focusing on the comparison against bulk SGE. Overall, the study is very interesting and highlights the need for human studies over model systems to assess these complex interactions. This study opens several follow up questions for researchers in the field to expand on and offers beneficial novel information on specific salivary protein therapeutic candidates.

Minor
Fig 3 and Fig 6 legend, Lu. longipalpus needs to be italicized.
Describe endotoxin test or source in the methods.
Add the protein concentration and time cultured before supernatants taken in Fig 4 legend.

Major
Please add some explanation of why plasma IgE and allergy status was assayed in cohort.
Line 129-133, there is mention about distal site reaction that implies the contralateral arm was used in a previous bite site weeks before. Does this mean the bite site arm was switched each administration? Or same area every bite administration? If the contralateral arm was previously bitten, it has implications for the biopsy results. Please clarify.
Was IHC performed on the contralateral biopsy? The % marker positive frequencies would be more meaningful if the contralateral % or counts/HPF were shown for comparison.
If more RNA/cDNA is available from the biopsies additional phenotyping transcripts could help identify cell types in the skin such as confirming CD4/CD8 ratio with CD3, neutrophil, plasma cell, NK and NKT markers.
Line 168, MPO staining is attributed to macrophages but is more highly expressed by neutrophils. Judging by the robust CD68+ cells in participant 1 and the lack of MPO, the MPO may not be secreted by macrophages. Neutrophils may be attracted to saliva or induced cytokines, they should be mentioned as potential infiltrate in this section.
Line 175, similarly, the increased IFNy mRNA in the skin biopsy is attributed to Th1 cells when there was no identification of T cells as the source of the transcript. The authors should consider using Type 1, Type 2, and Type 17 language over Th1, Th2, and Th17 when cellular sources of cytokines is not confirmed.
While reading I continuously questioned the choice of negative control for PBMC cytokine assays. I only found the apheresis comments at the very end and that is unfortunate but I understand why the choice was made to use other non-exposed control cells. However, the additional control that would make sense is an unstimulated group. Or stimulated with an irrelevant protein. This would allow analysis of whether markers were upregulated by individual proteins instead of only in comparison to SGE.
For the PBMC/infected macrophage co-culture, please add additional protocol information to the graphic including time of macrophage infection (8hrs), time of PBMC activation by salivary proteins (4hrs), and time of co-culture (5 days).
- Why was infection not quantified prior to co-culture? Each donor likely had a different infection level and the % reduction may be more significant in exposed donors.
- The time of macrophage infection is quite short. At this timepoint, internalized parasites will not have converted to amastigotes yet. Please highlight more about the design of the experimental timing.
The finding that the salivary proteins induced killing in cocultures from unexposed individuals could be related to the decision to use GM-CSF to differentiate monocyte derived macrophages and not M-CSF. GM-CSF is known to produce

macrophages with an inflammatory phenotype. So you may have pre-activated your macrophages before the experiment. This can be repeated with healthy donor PBMCs comparing GM-CSF and M-CSF differentiation to confirm.

Version 1:

Reviewer comments:

Reviewer #2

(Remarks to the Author)
I believe that the authors have responded to my comments satisfactorily.

Reviewer #3

(Remarks to the Author)
The updates to the manuscript figures and discussion have improved the strength of the conclusions and the clarity of the findings. No further revisions requested.

Open Access This Peer Review File is licensed under a Creative Commons Attribution 4.0 International License, which permits use, sharing, adaptation, distribution and reproduction in any medium or format, as long as you give appropriate credit to the original author(s) and the source, provide a link to the Creative Commons license, and indicate if changes were made.
In cases where reviewers are anonymous, credit should be given to 'Anonymous Referee' and the source.
The images or other third party material in this Peer Review File are included in the article's Creative Commons license, unless indicated otherwise in a credit line to the material. If material is not included in the article's Creative Commons license and your intended use is not permitted by statutory regulation or exceeds the permitted use, you will need to obtain permission directly from the copyright holder.
To view a copy of this license, visit https://creativecommons.org/licenses/by/4.0/

Response to Reviewers

Abdeladhim *et al*.
Manuscript #: COMMSBIO-25-9571

Reviewers' comments:

Reviewer #1 (Remarks to the Author):

This work is relevant to the field of leishmaniasis and provides useful evidence on the immune responses generated after repeated exposure of Lu. longipalpis bites in humans. Despite limitations aknowledged by the authors, the results contribute to the understanding of immunity generated by this sand fly species salivary proteins, identifiyng potential vaccine candidates and raising new questions.
The paper is well-written, the methodology fully described and the statistical analyses are valid.

Comment #1-1: *I have no comments on the manuscript, just suggest to review the use of italics for the vector species throughout the document.*

Response: We thank the reviewer for their positive feedback on the manuscript. We have reviewed the document and italicized all vector species names throughout.

Reviewer #2 (Remarks to the Author):

The study on controlled exposure to sand fly bites in humans is tremendous, providing many meaningful findings, and I found this worthy for being shared to a wide range of authors working in immunology, parasitology and entomology. At the same time, the study somewhat struggles with showing statistical significance to draw conclusions the authors would like to deliver. I understand the difficulty in having clear-cut data on clinical subjects, but careful interpretations and discussions of data as well as statements of limitations would help readers aware of the significance of the study, so the authors are encouraged to consider comments provided below.

Comment #2-1: *L169: 'The IHC data also suggest that CD4–CD8– T cells (termed double negative or DN T cells) may play a role in the DTH response to sand fly bites.' Though this claim is partly reasonable according to previous papers, technical problem /sensitivity issue on CD4 staining by IHC cannot be ruled out.*

Response: We appreciate the reviewer's concern and now explicitly acknowledge in the Discussion that IHC sensitivity limitations cannot be formally excluded.

Revised Lines 451-452: "While we cannot formally exclude the possibility of incomplete detection of CD4 by IHC, our report of DN T cells corroborates prior findings in a cohort from Mali…"

Based on our prior experience with this anti-CD4 antibody clone, however, we consider it unlikely that the low detection of CD4 in our IHC experiments reflects a technical issue. In our prior study of DTH reactions to sand fly bites in a naturally exposed Malian cohort using the same anti-CD4 antibody clone, 3 of 6 individuals exhibited strong $CD3^+$ T-cell infiltration with absent $CD4^+$ or $CD8^+$ staining (Oliveira *et al.*, DOI: 10.1038/jid.2012.315), similar to our current findings of DN T cells in Figure 3. Importantly, strong $CD4^+$ staining

was observed in other individuals in that cohort (Figure 2 of Oliveira *et al.*), demonstrating that this protocol can sensitively detect CD4 in FFPE skin sections.

Comment #2-2: *For example, Ref. 20 surely describes the presence of DN T cells in CL lesions, the proportion is much less than CD4+ T cells. Ref. 21 reports a higher proportion of DN T cells, whereas CD4+ T cells still account for one third of T cell population. As an accumulation of CD4+ T cells is a hallmark of DTH, careful interpretation of the data is necessary if the authors would like to claim the observed reaction is truly DTH.*

Response: The reviewer raises the important question of whether the observed reaction represents a true DTH response. Classical DTH responses, such as the tuberculin or leishmanin skin tests, are typically characterized by $CD4^+$ T cell accumulation. However, subsequent reports have also described DTH reactions composed predominantly of $CD8^+$ T cells, with $CD4^+$ cells largely absent by IHC (Zeltser *et al.*, DOI: 10.1001/archderm.137.7.893; Kalish and Askenase, DOI: 10.1016/S0091-6749(99)70489-6). In these studies, DTH was defined by reaction kinetics (24-48 hours post-exposure) and the predominance of T cells and macrophages within the infiltrate, rather than by $CD4^+$ T cell abundance alone.

Because the reactions we observed meet these kinetic and cellular criteria, but with a T cell infiltrate composed largely of DN and $CD8^+$ T cells, and appreciating the reviewer's point regarding the well-described classical DTH reaction, we have now defined them as a "non-classical" DTH response. This terminology reflects our interpretation of these reactions as DTH responses by broader immunological criteria, while distinguishing them from the $CD4^+$ T cell-dominated classical DTH observed in TST or LST.

Revised Lines 175-179: "The IHC data also suggest that $CD4^-CD8^-$ T cells (termed double negative or DN T cells) may play a role in the DTH response to sand fly bites. The low frequency of CD4+ T cells suggests these reactions represent what we have defined as a "non-classical" DTH response where non-CD4+ T cell subsets predominate, similarly to what has been described in other skin pathologies."

Revised Lines 448-450: "Notably, both biopsies showed a relative absence of CD4+ T cells, and were instead dominated by CD3+ CD4– CD8– double negative (DN) T cells, consistent with a non-classical DTH phenotype."

Comment #2-3: *One of the potential solutions which can be done using obtained samples is to assess CD4 gene expression using RNA isolated from the biopsy samples. If performing additional experiments is difficult, at least limitations need to be discussed in the paragraph starting on L317.*

Response: Unfortunately, no additional RNA or cDNA is available from these specimens. We have added comments in the Discussion highlighting the need for future studies to more precisely define infiltrating cell subsets at the bite site in humans, including DN T cells.

Revised Lines 459-463: "Further studies utilizing RNA-seq and flow cytometry are needed to better define the immune cell subsets infiltrating the sand fly bite site in humans, to elucidate the dominant T_H polarization profile at the sand fly bite site, and to determine whether DN T cells are a consistent and prominent feature of the DTH response that influences immunity to *Leishmania* parasites."

Comment #2-4: *Fig. 4a and 5a: Though I understand a difference in measurement methods, why do IFN-γ levels differ this much (almost two orders when medians are compared) between the two experiments?*

Response: We appreciate the reviewer's concern; although we cannot fully account for the magnitude of the difference, we believe it primarily reflects technical differences between Luminex and ELISA platforms. In addition, for salivary gland extract (SGE), which showed the largest discrepancy, we have informally observed marked batch-to-batch variability in immunogenicity, likely due to unavoidable variation in gland dissection and processing. Because all comparisons were made only within samples analyzed using the same assay, we believe the conclusions drawn from Figures 4 to 6 remain valid.

Comment #2-5: Fig. 5: *It is new to me to interpret log2 cytokine concentration ratio of 0 as a boarder for Th1/Th2. I do not think this is a well-accepted method.*

Response: We appreciate the reviewer's concern and have revised the Methods and Results to clarify both the rationale and interpretation of the type 1/type 2 cytokine ratio analyses. Log-transformed cytokine ratios have been previously used to examine relative type 1 versus type 2 cytokine balance in human immune responses (e.g., Mihret *et al*., DOI: 10.1186/1471-2334-14-125), as the log scale stabilizes variance and reduces the influence of extreme right-skew commonly observed in cytokine data.

In our analysis, cytokine concentrations were first background-subtracted from values treated with media only for each cytokine using participant-matched PBMCs. Cytokine ratios were then calculated from these background-corrected values. As a result, the media-only condition is mathematically centered at zero on the log2 ratio scale by design. For Figures 5b-5g, deviation from zero therefore reflects a stimulation-induced shift in the relative balance of type 1 versus type 2 cytokines within individuals, rather than an absolute polarization threshold.

Statistical significance was assessed using the Wilcoxon signed-rank test, with the null hypothesis that stimulation does not shift the cytokine ratio relative to the media-only baseline. Accordingly, the magnitude of deviation from zero reflects a consistent population-level shift away from baseline. We have revised the Methods, Results, and figure legends to emphasize that these ratios represent relative within-study comparisons to the media-treated baseline (Lines 234-235, 241, 281-282, 286-288, 834-836, 867-871, 877-884).

Comment #2-6: *In general, IFN-γ shows a higher concentration than IL-4 or IL-5, and if sole concentration ratio is used, it naturally shows a trend of 'Th1-biased immunity' in many cases.*

Response: We acknowledge that IFN-γ is often detected at higher concentrations than IL-4 or IL-5, however this pattern was not uniformly observed in our dataset. SGE-treated PBMCs showed a mixed type 1/type 2 response across most cytokine pairs, whereas LJL143 stimulation resulted in a significantly higher IFN-γ/IL-5 ratio over baseline (Fig. 5d, $p < 0.001$). Thus, even in the context of higher absolute IFN-γ concentrations, this approach allows relative mixed and type 1-polarized responses to be distinguished in our dataset.

Comment #2-7: *Besides, IFN-γ/IL-17 is no longer Th1/Th2.*

Response: We agree and have removed IFN-γ/IL-17 and IL-12/IL-17 ratios from Figure 5 and Supplementary Figure 3 (Line 303), relocating these data to new Supplementary Figure 2.

Revised Figure 5 with IL-17 data removed in 5b-g:

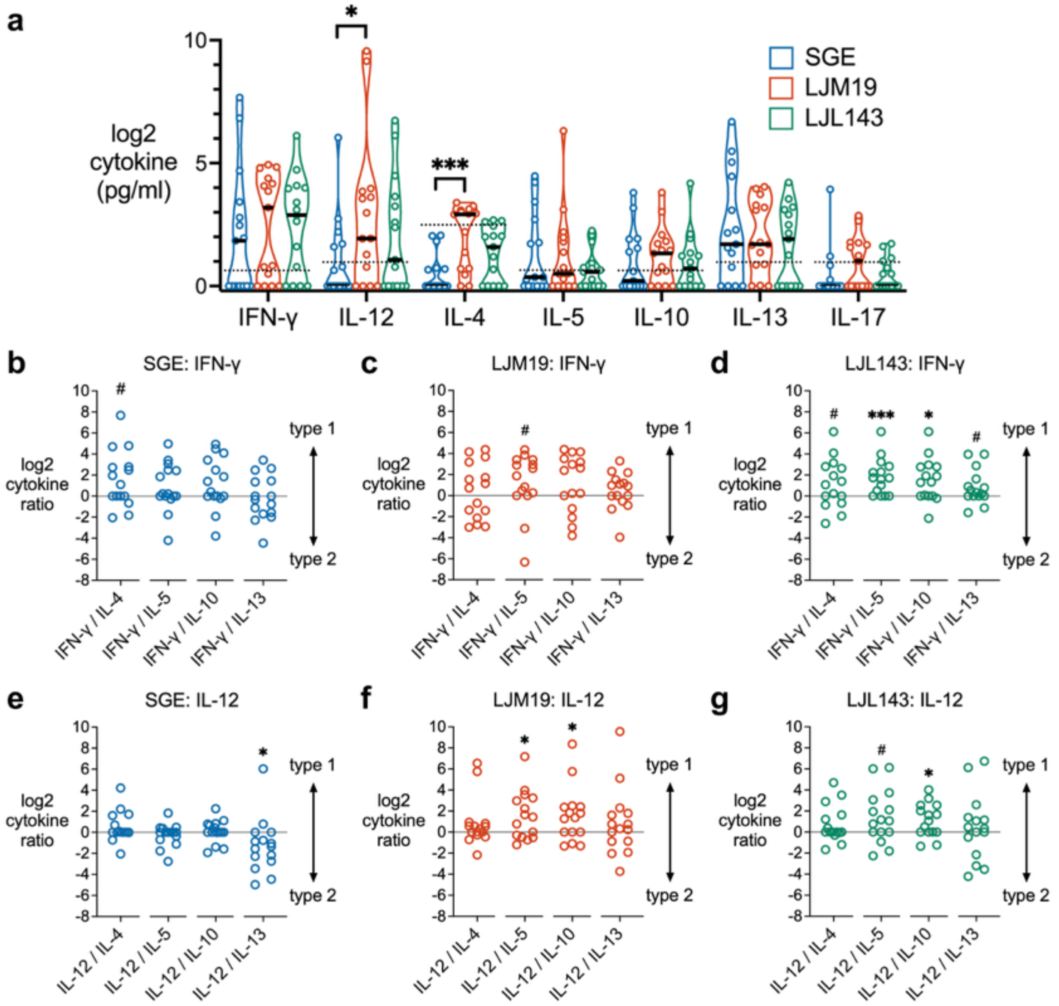

New Figure S2:

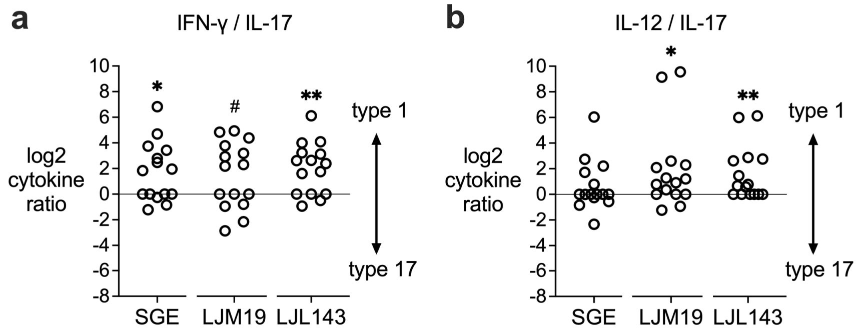

Revised Figure S3 (previously Figure S2) with IL-17 data removed:

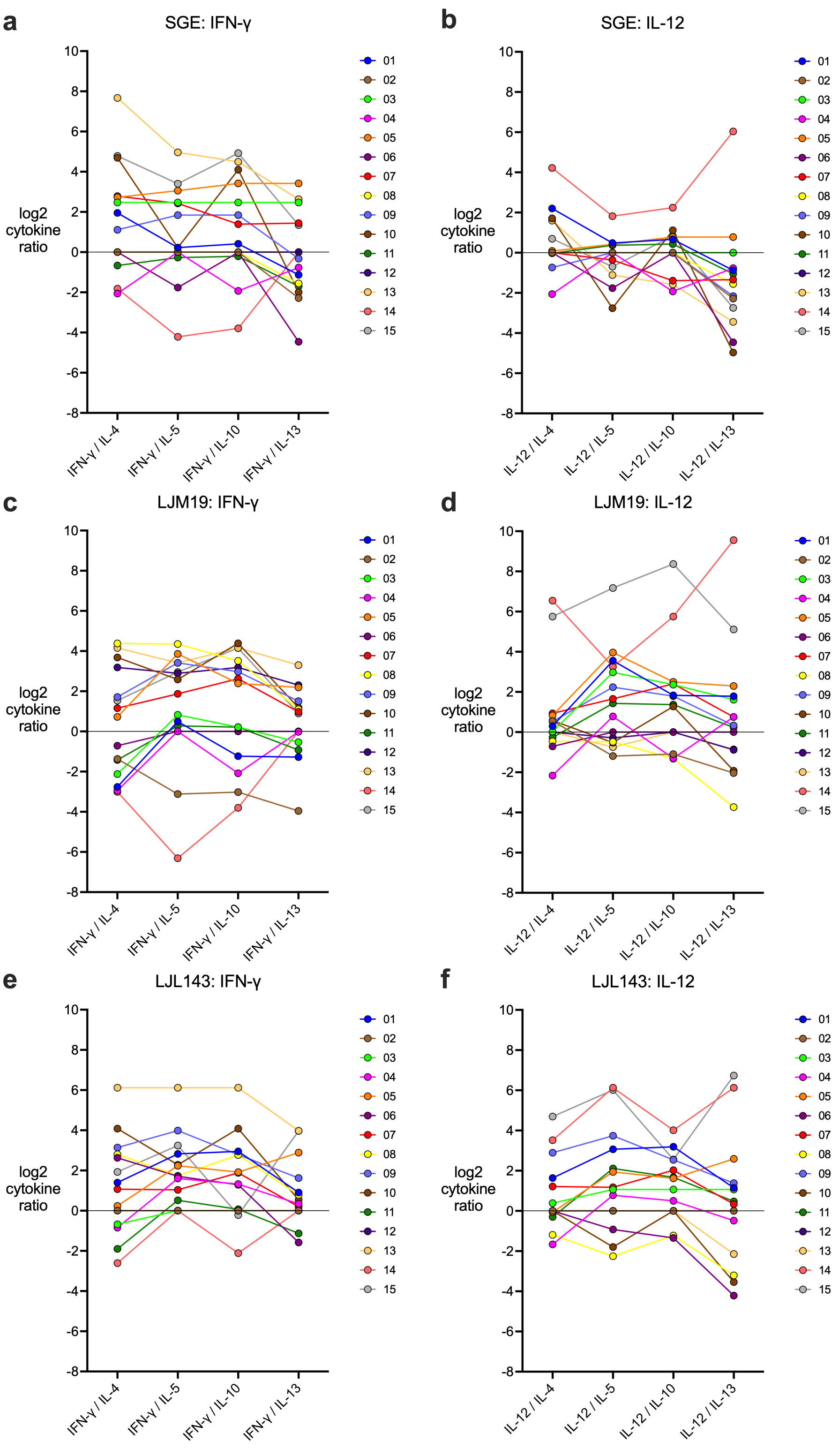

Comment #2-8: *I am also confused on the authors' intention on the figure. If the intention is to claim 'LJM19 and LJL143 induce a TH1-polarized cytokine response (L194)', the authors need to do comparison between those defined antigens and SGE, as they did for Fig. 5a. Despite that, Fig. 5b-g do not involve statistical analyses among antigens. Proper analyses are required to draw conclusions the authors would like to claim.*

Response: The analyses in Figures 5b-5g were designed to assess polarization relative to participant-matched background cytokine secretion, represented by zero on the log scale. In contrast, Figure 5a addresses a distinct question that answers the reviewer's question of whether individual cytokine production differs between salivary proteins and SGE. We have revised the Results to clarify these distinct analytical goals.

Revised Lines 235-236: "Comparing the three stimulation conditions for each cytokine, we found that…"

Revised Lines 239-241: "To quantify type 1/type 2 cytokine polarization induced by SGE or each salivary protein, we calculated the ratios of the type 1 cytokines, IFN-γ and IL-12, against each type 2 cytokine (IL-4, IL-5, IL-10, and IL-13) and the type 17 cytokine, IL-17 relative to media-treated controls."

Comment #2-9: *Fig. 7b and Fig. S3: Can you enumerate 'the number of amastigotes per macrophages (both infected and uninfected)' and see if there is any statistical difference between the unexposed and exposed? This is proposed because no statistical difference was observed between the unexposed and exposed in Fig. 7b or Fig. S3;*

Response: We thank the reviewer for this insightful suggestion, as the additional analysis clarified the distinct contributions of innate and adaptive immunity to parasite killing. We have added this new analysis as Figure 7c, updated the Methods section, and removed Figure S3 to avoid redundancy.

Revised Lines 894-898: "The number of amastigotes per macrophage was found to have a lognormal distribution with no significant differences in variance between treatment groups. Thus, we used an unpaired lognormal t-test to compare the number of amastigotes per macrophage between unexposed and exposed samples for LJM19 or LJL143."

By quantifying amastigote burden per macrophage (reported as amastigotes per 100 macrophages for clarity), we observed that stimulation with LJM19 or LJL143 significantly reduced amastigote load relative to media-treated controls only in exposed individuals. In unexposed individuals, these reductions did not reach statistical significance (vs. media: $p = 0.35$ for LJM19; $p = 0.22$ for LJL143). The observed interindividual variability may have reduced statistical power in these between-subject comparisons. In contrast, within-group participant-matched comparisons were more consistent, allowing detection of treatment-specific effects. Taken together, these data suggest that prior sand fly exposure is associated with enhanced parasite-killing effects of LJM19 and LJL143 beyond their innate immune-activating activity, primarily by reducing intracellular amastigote burden (Lines 443-457).

Revised Figure 7 with revised panel (a) and new panel (c):

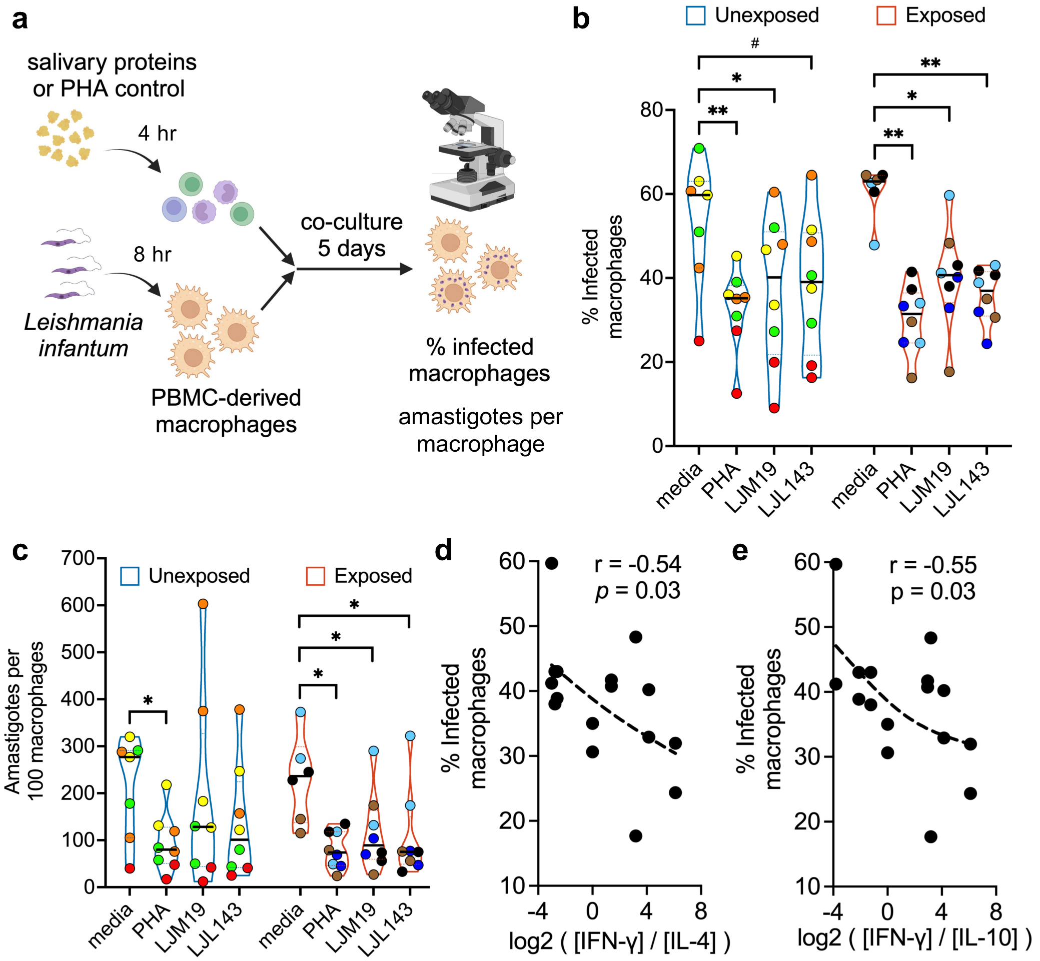

Comment #2-10: *'a narrower data spread' (L272) is not very helpful to evoke the presence of antigen-specific Th1 cells limiting parasite infection.*

Response: We agree with this critique and have removed this statement from the Discussion.

Comment #2-11: *If none of the analysis does not work, limitations are better to be discussed more carefully in the Discussion section. For example, L362 says 'our data from human PBMCs show that LJL143 promotes the killing of L. infantum by macrophages ex vivo.' in the context of vaccines, i.e., antigen-specific acquired immunity. But, the data say the parasite-killing activity of LJL143 is not depended on antigen-specific acquired immunity. This kind of overinterpretation can also be found elsewhere (L399 – 411).*

Response: The new analysis presented in Figure 7c directly addresses this concern. Analysis of the percentage of infected macrophages (Figure 7b) shows that, in unexposed individuals, parasite killing attributable to innate immune activation is evident for LJM19 and trends toward significance for LJL143. In contrast, the amastigote burden analysis in Figure 7c reveals a further reduction in parasite load following sand fly exposure, consistent with the development of adaptive anti-saliva immunity.

We have revised the Results and Figure 7 legend to reflect these distinctions more clearly (Lines 387-388, 401-415). In addition, we have substantially revised and reorganized the Discussion to emphasize the innate, adjuvant-like properties of LJM19 and LJL143 (Lines 505-509, 518-522), and to clarify that their enhancement of anti-*Leishmania* immunity likely reflects the combined action of innate immune activation and exposure-dependent adaptive responses, rather than antigen-specific adaptive immunity alone (Lines 523-524, 626-628). We thank the reviewer for this critique, which strengthened the interpretation and framing of our findings.

Reviewer #3 (Remarks to the Author):

Summary
This study established a cohort of 15 sand fly saliva immunized humans, previously unexposed, through repeated exposure over the course of 46 weeks. This resulted in a cohort with salivary protein reactive cellular response and presumed skin tissue resident immune seeded environment. The authors assayed the site of skin exposure for high level skin infiltration post challenge in 2 subjects, which showed highly variable infiltrate, but both indicated a significant presence of double negative T cells in the skin, which is an interesting finding to follow up on as DN T cells are underexplored. The authors also assays PBMCs restimulated with salivary proteins to identify the two specific proteins that induce the strongest Type 1 polarized response. These proteins are able to induce antigen-specific IFNy secretion at levels similar to whole salivary gland extract. This is a considerable achievement as bulk SGE has variable pleiotropic and non-specific effects on local cells whereas use of a specific recombinant protein has more potential for therapeutic development and controlled testing of effects. Finally, a coculture experiment using autologous parasite infected macrophages and salivary protein stimulated PBMCs shows that salivary protein stimulated PBMCs results in significantly fewer infected macrophages compared to unstimulated PBMCs. This finding is to be determined if it is influenced on pre-immunization with salivary proteins or not. The study lacks flow cytometry on the antigen stimulated PBMCs to phenotype relevant cytokine secreting cell type lineage and memory/effector phenotype. The study also lacks unstimulated controls, instead focusing on the comparison against bulk SGE. *Overall, the study is very interesting and highlights the need for human studies over model systems to assess these complex interactions. This study opens several follow up questions for researchers in the field to expand on and offers beneficial novel information on specific salivary protein therapeutic candidates.*

Response: We thank the reviewer for their thoughtful and positive summary of our work.

Minor

<u>Comment #3-1</u>: *Fig 3 and Fig 6 legend, Lu. longipalpis needs to be italicized.*

Response: We have italicized *Lu. longipalpis* in the Figure 3 and Figure 6 legends (Lines 165, 342).

Comment #3-2: *Describe endotoxin test or source in the methods.*

Response: We have added the endotoxin test kit and manufacturer information to the Methods section.

Revised Lines 786-787: "Endotoxin levels were measured with the Endosafe Portable Test System (Charles River Laboratories) and verified to be <20 EU/ml for all proteins."

Comment #3-3: *Add the protein concentration and time cultured before supernatants taken in Fig 4 legend.*

Response: This information has been added to the Figure 4 legend.

Revised Line 226: "PBMCs were stimulated with salivary gland extract (SGE) or the indicated recombinant Lu. longipalpis salivary protein (10 µg/ml) for 96 hours to measure IFN-γ levels by ELISA."

Major

Comment #3-4: *Please add some explanation of why plasma IgE and allergy status was assayed in cohort.*

Response: We have added an explanation of the rationale for assessing plasma IgE and allergy status to the Results section.

Revised Lines 98-99: "Volunteers were screened for allergy status and plasma IgE to exclude individuals potentially at higher risk for developing severe allergic reactions to sand fly bites."

Comment #3-5: *Line 129-133, there is mention about distal site reaction that implies the contralateral arm was used in a previous bite site weeks before. Does this mean the bite site arm was switched each administration? Or same area every bite administration? If the contralateral arm was previously bitten, it has implications for the biopsy results. Please clarify.*

Response: Feeding sites were alternated between arms on consecutive visits (Line 815). Thus, the contralateral biopsy site may have been previously bitten but was free of visible inflammation at the time of biopsy. For this reason, we refer to these samples as "normal appearing skin free of visible inflammation on the contralateral arm" (Lines 143-144, 724-725) and do not describe them as "unbitten skin."

Comment #3-6: *Was IHC performed on the contralateral biopsy? The % marker positive frequencies would be more meaningful if the contralateral % or counts/HPF were shown for comparison.*

Response: IHC was not performed on contralateral biopsies because no cellular infiltrate was observed by H&E staining. Accordingly, IHC analyses were restricted to bite site biopsies to characterize the cellular composition of the bite-induced infiltrate.

<u>Comment #3-7</u>: *If more RNA/cDNA is available from the biopsies additional phenotyping transcripts could help identify cell types in the skin such as confirming CD4/CD8 ratio with CD3, neutrophil, plasma cell, NK and NKT markers.*

Response: Unfortunately, no additional RNA or cDNA is available from these specimens. We have added comments in the Discussion highlighting the importance of future studies incorporating RNA-seq and flow cytometry to define infiltrating cell populations in greater detail (Lines 459-463).

<u>Comment #3-8</u>: *Line 168, MPO staining is attributed to macrophages but is more highly expressed by neutrophils. Judging by the robust CD68+ cells in participant 1 and the lack of MPO, the MPO may not be secreted by macrophages. Neutrophils may be attracted to saliva or induced cytokines, they should be mentioned as potential infiltrate in this section.*

Response: We thank the reviewer for highlighting this ambiguity. We have clarified the manuscript text to explicitly state that MPO is used here as a marker of neutrophils, not macrophages.

Revised Lines 163, 172-173: "While Participant #1 had no detectable neutrophils by myeloperoxidase (MPO) staining (Fig. 3c), Participant #13 showed neutrophil infiltration throughout the dermis (Fig. 3d)."

<u>Comment #3-9</u>: *Line 175, similarly, the increased IFNy mRNA in the skin biopsy is attributed to Th1 cells when there was no identification of T cells as the source of the transcript. The authors should consider using Type 1, Type 2, and Type 17 language over Th1, Th2, and Th17 when cellular sources of cytokines is not confirmed.*

Response: We agree with this recommendation and have revised the manuscript throughout to use "Type 1," "Type 2," and "Type 17" terminology when cellular sources of cytokines are not directly identified.

<u>Comment #3-10</u>: *While reading I continuously questioned the choice of negative control for PBMC cytokine assays. I only found the apheresis comments at the very end and that is unfortunate but I understand why the choice was made to use other non-exposed control cells. However, the additional control that would make sense is an unstimulated group. Or stimulated with an irrelevant protein. This would allow analysis of whether markers were upregulated by individual proteins instead of only in comparison to SGE.*

Response: We appreciate the reviewer's understanding of the technical challenges we encountered with apheresis in our pre-exposure PBMC collections. As with the ELISA data (Figure 4), multiplex cytokine data (Figures 5 and 6) represent cytokine concentrations induced by SGE, LJM19, or LJL143 following background subtraction using participant-matched PBMCs treated with media only. We have clarified this normalization approach in the Methods, Results, and figure legends (Lines 234-235, 241, 281-282, 286-288, 834-836, 867-871, 877-884).

While inclusion of non-immunogenic salivary proteins as additional controls would have been informative, this was not feasible due to the limited volume of PBMCs collected per participant, necessitating prioritization of the most immunogenic candidates, LJM19 and LJL143.

Comment #3-11: *For the PBMC/infected macrophage co-culture, please add additional protocol information to the graphic including time of macrophage infection (8hrs), time of PBMC activation by salivary proteins (4hrs), and time of co-culture (5 days).*

Response: We have added the requested protocol details to the experimental schematic in Figure 7a (see revised version of Figure 7 above).

Comment #3-12: *Why was infection not quantified prior to co-culture? Each donor likely had a different infection level and the % reduction may be more significant in exposed donors.*

Response: Infection was not quantified prior to co-culture because parasite burden was assessed by Giemsa staining, which requires cell fixation. Interindividual variation in baseline infection is illustrated in new Figure 7c using media-only controls. For this reason, treatment effects were evaluated using participant-matched comparisons within exposure groups, accounting for baseline differences. Significant reductions in amastigote load were observed only in exposed donors, as reflected in new Figure 7c (see revised version of Figure 7 above).

Comment #3-13: *The time of macrophage infection is quite short. At this timepoint, internalized parasites will not have converted to amastigotes yet. Please highlight more about the design of the experimental timing.*

Response: We thank the reviewer for raising this point. The experimental timing was designed to capture early events following parasite entry into macrophages, prior to amastigote differentiation, during the time window when salivary protein vaccines would be expected to exert their immunologic effects to influence parasite killing. We have clarified this rationale in the Results.

Revised Lines 330-333: "We sought to capture the effects of LJM19 and LJL143 on early macrophage infection by Leishmania, because this is the time window when the proteins would begin to enhance parasite killing as candidate vaccine antigens and adjuvants."

Comment #3-14: *The finding that the salivary proteins induced killing in cocultures from unexposed individuals could be related to the decision to use GM-CSF to differentiate monocyte derived macrophages and not M-CSF. GM-CSF is known to produce macrophages with an inflammatory phenotype. So you may have pre-activated your macrophages before the experiment. This can be repeated with healthy donor PBMCs comparing GM-CSF and M-CSF differentiation to confirm.*

Response: We agree that GM-CSF differentiation polarizes macrophages toward an inflammatory phenotype and have added this limitation to the Discussion. However, because all macrophages were differentiated under identical conditions, comparisons across treatments remain internally consistent (Figure 7b). The additional reduction in amastigote burden observed only in exposed individuals (Figure 7c) further supports an exposure-dependent enhancement of parasite killing.

Revised Lines 617-621: "Due to their differentiation in GM-CSF, the PBMC-derived macrophages in the parasite killing assay were likely polarized to the inflammatory M1 phenotype,59 which would enhance their baseline ability to kill intracellular pathogens. Nevertheless, because all macrophages were differentiated under identical conditions, comparisons across treatments remain internally consistent."
